# Supplementary material for: Causal interplay between lactose intolerance and gut microbiota: a combined bidirectional Mendelian randomization and in vivo validation study
Source: Front Nutr. 2026 Jun 1;13:1803337. doi: 10.3389/fnut.2026.1803337 (PMC13265576; doi:10.3389/fnut.2026.1803337)
Supplement: Supplementary file 1 [file Data_Sheet_1.zip › supplementary materials/Forward/leave one out cross validation/ebi-a-GCST90027737.finngen_R12_E4_LACTONAS.pdf]

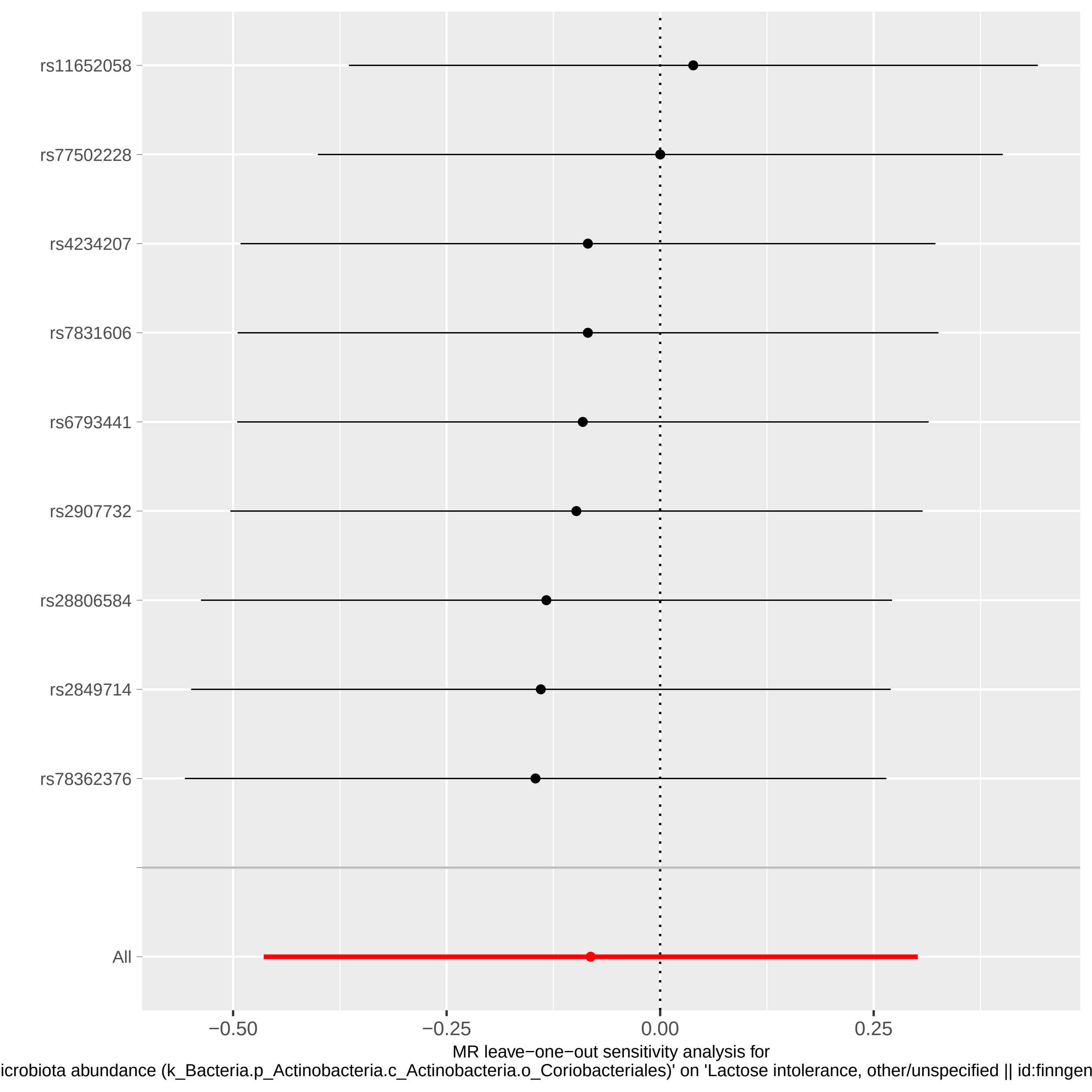
rs11652058
rs77502228
rs4234207
rs7831606
rs6793441
rs2907732
rs28806584
rs2849714
rs78362376
All
−0.50
−0.25
0.00
0.25
MR leave−one−out sensitivity analysis for
icrobiota abundance (k_Bacteria.p_Actinobacteria.c_Actinobacteria.o_Coriobacteriales)' on 'Lactose intolerance, other/unspecified || id:finngen
